# Electrically Tunable Fano Resonance from the Coupling between Interband Transition in Monolayer Graphene and Magnetic Dipole in Metamaterials

**DOI:** 10.1038/s41598-017-17394-y

**Published:** 2017-12-07

**Authors:** Bo Liu, Chaojun Tang, Jing Chen, Mingwei Zhu, Mingxu Pei, Xiaoqin Zhu

**Affiliations:** 1 School of Mathematics and Physics, Jiangsu University of Technology, Changzhou, 213001 China; 2 COOR, Collaborative Innovation Center for Information Technology in Biological and Medical Physics, College of Science, Zhejiang University of Technology, Hangzhou, 310023 China; 3 College of Electronic and Optical Engineering & College of Microelectronics, Nanjing University of Posts and Telecommunications, Nanjing, 210023 China; 4 State Key Laboratory of Millimeter Waves, Southeast University, Nanjing, 210096 China; 5 National Laboratory of Solid State Microstructures and Department of Materials Science and Engineering, Nanjing University, Nanjing, 210093 China

## Abstract

Fano resonance modulated effectively by external perturbations can find more flexible and important applications in practice. We theoretically study electrically tunable Fano resonance with asymmetric line shape over an extremely narrow frequency range in the reflection spectra of metamaterials. The metamaterials are composed of a metal nanodisk array on graphene, a dielectric spacer, and a metal substrate. The near-field plasmon hybridization between individual metal nanodisks and the metal substrate results into the excitation of a broad magnetic dipole. There exists a narrow interband transition dependent of Fermi energy *E*_*f*_, which manifests itself as a sharp spectral feature in the effective permittivity *ε*_*g*_ of graphene. The coupling of the narrow interband transition to the broad magnetic dipole leads to the appearance of Fano resonance, which can be electrically tuned by applying a bias voltage to graphene to change *E*_*f*_. The Fano resonance will shift obviously and its asymmetric line shape will become more pronounced, when *E*_*f*_ is changed for the narrow interband transition to progressively approach the broad magnetic dipole.

## Introduction

Fano resonance in a variety of plasmonic nanostructures and metamaterials has been drawing a lot of attentions in the past decade^1–4^, thanks to its great potential for many applications such as refractive-index sensing or biosensing^5–12^, surface-enhanced Raman scattering (SERS)^13^, light emission enhancement^14,15^, nonlinear optical process^16,17^, slowing light^18^, and so on. In order to obtain Fano resonance, breaking the geometric symmetry has been widely explored for a sharp subradiant (dark) mode to be excited and then interact with a broad superradiant (bright) mode^5–8,19,20^, although symmetry breaking is not mandatory for Fano resonance in some nanostructures^21,22^. Recently, there has been increasing interest in dynamically tunable Fano resonance whose resonance wavelength and excitation strength can be changed by internal or external parameters, because tunable Fano resonance can find more flexible and important applications in practice. A very effective approach to tune Fano resonance is directly adjusting the structural parameters of plasmonic nanostructures and metamaterials^23–26^. Fano resonance can also be tuned by external mechanical stress^27^, voltages^28^, temperature^29^, and magnetic field^30^. Furthermore, some studies have demonstrated all-optical tunable Fano resonance by operating light pump intensity^31–33^.

Monolayer graphene exhibits intriguing electronic, optical, and mechanical properties, and is promising in optoelectronics, photonics, plasmonics, and metamaterials^34^. Especially, the complex surface conductivity of graphene can be rapidly and dramatically changed by electric gating, which has been utilized for realizing electronically tunable Fano resonance in metallic nanostructures and metamaterials with monolayer graphene integrated therein^35–41^. Moreover, very similar to noble metals, graphene in itself is able to support surface plasmon resonances (SPRs) in the mid-infrared and terahertz (THz) regime. Therefore, very recently there also have been many efforts to realize electronically tunable Fano resonance by carefully engineering graphene into nanostructures with various morphologies^42–51^.

It is well known that naturally occurring materials exhibit the saturation of the magnetic response beyond the THz regime. In the absence of natural magnetism, most Fano resonances reported so far are mainly based on purely electric effect. However, in the past several years there is also increasing interest in Fano resonance based on magnetic effects in metamaterials^52–59^. But, up to now there are only few researches on electronically tunable Fano resonance by graphene based on magnetic effect^60^.

We will theoretically study electrically tunable Fano resonance with asymmetric line shape over an extremely narrow frequency range in the reflection spectra of metamaterials. The metamaterials are composed of a metal nanodisk array on graphene, a dielectric spacer, and a metal substrate. The near-field plasmon hybridization between individual metal nanodisks and the metal substrate results into the excitation of a broad magnetic dipole. There exists a narrow interband transition dependent of Fermi energy *E*_*f*_, which manifests itself as a sharp spectral feature in the effective permittivity *ε*_*g*_ of graphene. The coupling of the narrow interband transition to the broad magnetic dipole leads to the appearance of Fano resonance, which can be electrically tuned by applying a bias voltage to graphene to change *E*_*f*_. The Fano resonance will shift obviously and its asymmetric line shape will become more pronounced, when *E*_*f*_ is changed for the narrow interband transition to progressively approach the broad magnetic dipole.

## Results

**Figure 1:**
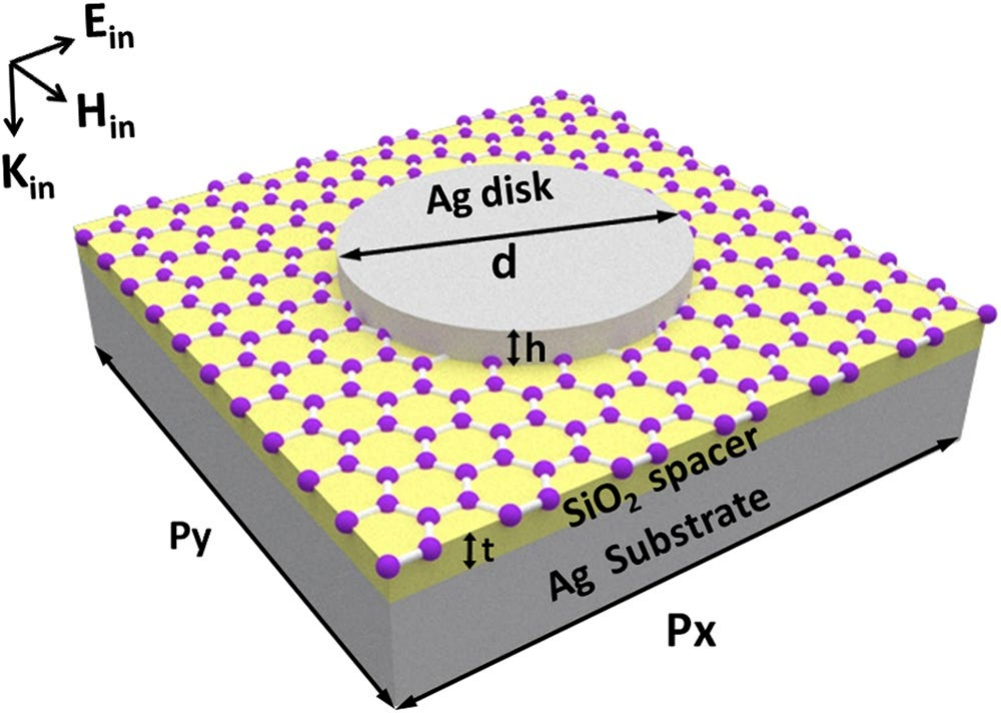
The designed metamaterials for electrically tunable Fano resonance. The period in the *x* (*y*) directicon is *p*_*x*_ (*p*_*y*_). The SiO_2_ spacer has a thickness of *t*. The Ag nanodisk has a diameter of *d* and a height of *h*. Light is normally incident, with a polarization along the *x* direction.

The designed metamaterials for electrically tunable Fano resonance are schematically shown in Fig. 1, which consist of an Ag nanodisk array on a graphene sheet, a SiO_2_ spacer, and an Ag substrate. In this work, the reflection and absorption spectra, and the distributions of electromagnetic fields are calculated by “EastFDTD, version 5.0”^61^. The refractive index of SiO_2_ is 1.45, and experimental data are used for the permittivity of Ag^62^. Graphene has a complex surface conductivity *σ*, which is the sum of intraband term *σ*_intra_ and interband term *σ*_*inter*_^63,64^, expressed as follow:

$${\sigma }_{{\rm{intra}}}=\frac{i{e}^{2}{k}_{B}T}{\pi {\hslash }^{2}(\omega +i/\tau )}(\frac{{E}_{f}}{{k}_{B}T}+2\,\mathrm{ln}({e}^{-\frac{{E}_{f}}{{k}_{B}T}}+1)),{\sigma }_{{\rm{inter}}}=\frac{i{e}^{2}}{4\pi \hslash }\,\mathrm{ln}(\frac{2{E}_{f}-(\omega +i/\tau )\hslash }{2{E}_{f}+(\omega +i/\tau )\hslash }).$$

In the above expression, *ω* is light frequency, *e* is electron charge, *ħ* is reduced Planck constant, *E*_*f*_ is Fermi energy, *τ* is relaxation time, *k*_*B*_ is Boltzmann constant, and *T* is temperature. The effective permittivity *ε*_*g*_ of graphene could be written as *ε*_*g*_ = 1 + *iσ*/(*ε*_0_ *ωt*_*g*_), where *ε*_0_ is vacuum permittivity, and *t*_*g*_ is graphene thickness.

**Figure 2:**
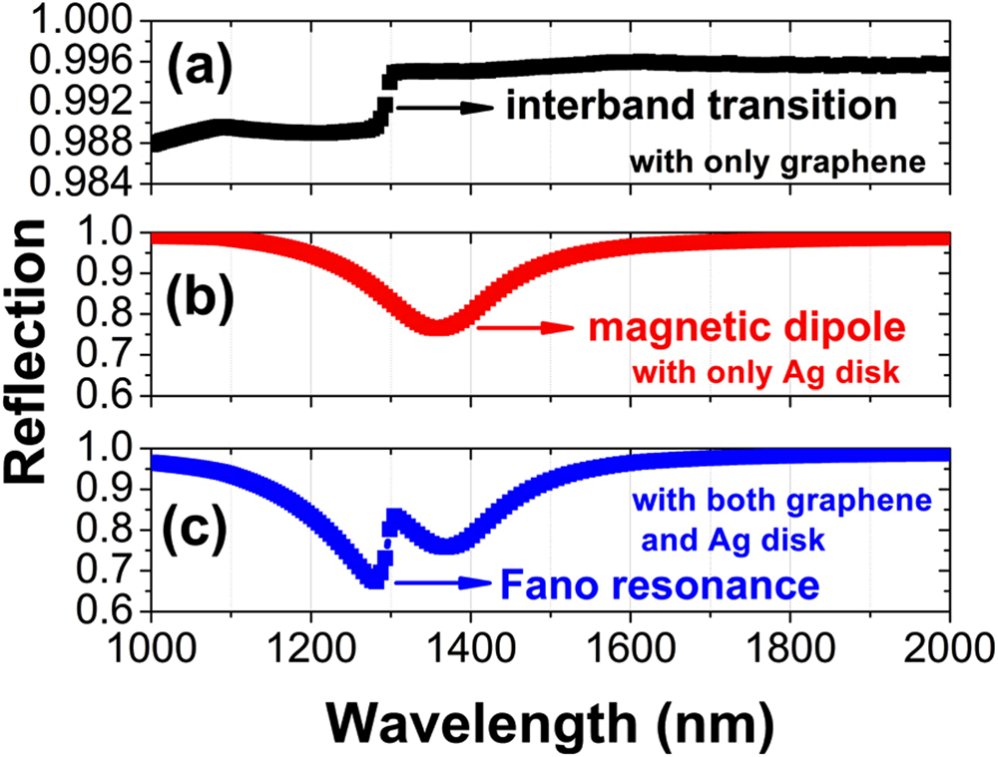
Calculated reflection spectra of metamaterials at normal incidence with only monolayer graphene (**a**), only Ag disk (**b**), and both monolayer graphene and Ag disk (**c**), as schematically shown in Fig. 1. Geometrical and physical parameters: *d* = 300 nm, *h* = 50 nm, *t* = 30 nm, *p*_*x*_ = *p*_*y*_ = 500 nm, *E*_*f*_ = 0.48 eV, *τ* = 0.50 ps, *T* = 300 K, *t*_*g*_ = 0.35 nm.

Figure 2(a) presents the reflection spectra in the case of only monolayer graphene at the SiO_2_ surface (i.e., the remainder after removing the Ag nanodisk in Fig. 1). In the reflection spectra, one can see an abrupt change around 1300 nm, which results from the narrow interband transition in the monolayer graphene. Figure 2(b) shows the normal-incidence reflection spectra, in the case of only Ag nanodisks at the surface of the SiO_2_ spacer supported on the Ag substrate (i.e., the remainder after removing the graphene in Fig. 1). A broad reflection dip centered at 1357 nm is observed, due to the excitation of magnetic dipole from the near-field plasmon hybridization between individual Ag nanodisks and the Ag substrate. Figure 2(c) shows the normal-incidence reflection spectra of metamaterials schematically shown in Fig. 1. In this case, Fano resonance appears and exhibits a pronounced asymmetric line shape with a sharp feature over an extremely narrow wavelength range near 1300 nm in the reflection spectra. The appearance of Fano resonance is from the coupling between the narrow interband transition in graphene and the broad magnetic dipole in metamaterials.

## Discussion

**Figure 3:**
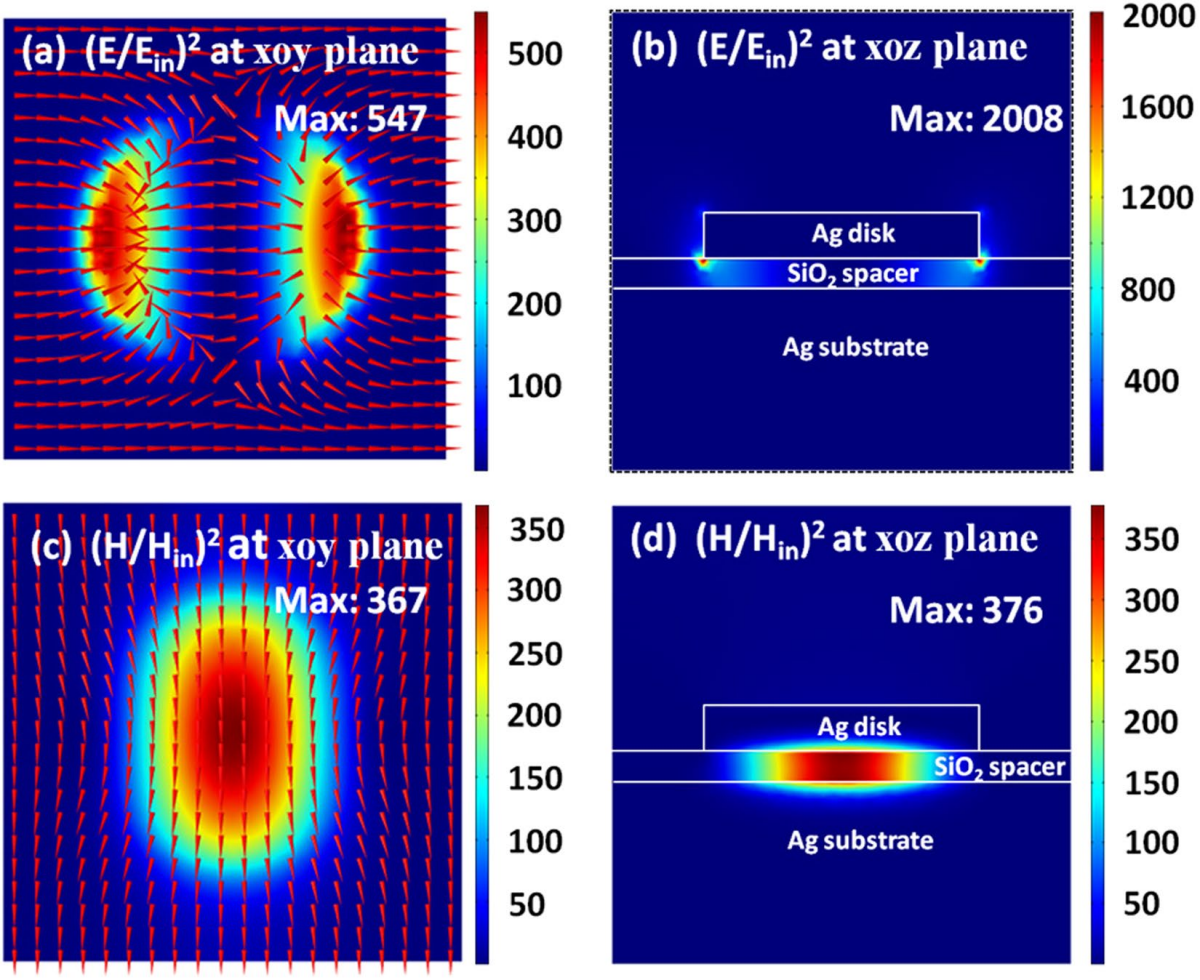
Electromagnetic field distributions on the *xoy* (**a**,**c**) and *xoz* (**b**,**d**) planes, at the resonance wavelength of magnetic dipole. The directions of electromagnetic fields are represented by the red arrows in (**a**,**c**). White lines in (**b**,**d**) outline the boundaries of different regions.

**Figure 4:**
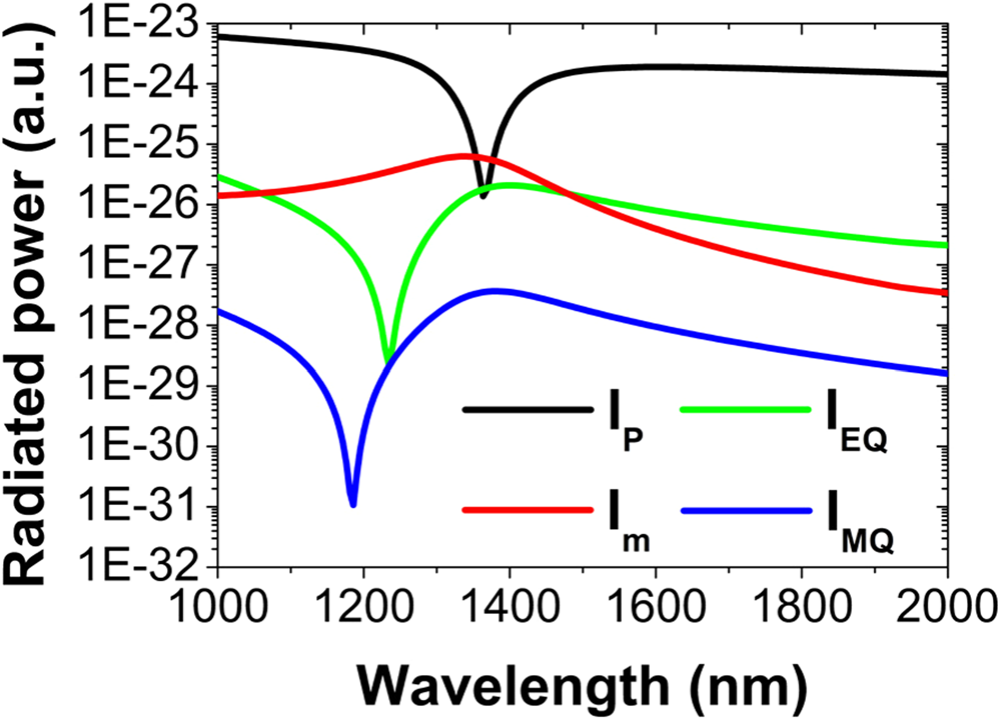
Radiated power from electric dipolar moment *I*_***p***_, magnetic dipolar moment *I*_***m***_, electric quadrupole dipolar moment *I*_***EQ***_, and magnetic quadrupole dipolar moment *I*_***MQ***_.

To confirm that the broad reflection dip in Fig. 2(b) is because of the excitation of a magnetic dipole in metamaterials, Fig. 3 plots the distributions of electromagnetic fields at the resonance wavelength of 1357 nm. The electric fields have two obvious “hotspots” in the vicinity of the nanodisk (see Fig. 3(a) and (b)), and the magnetic fields have a “hotspot” between the nanodisk and the substrate (see Fig. 3(c) and (d)). Such a field distribution is closely related to the excitation of a magnetic dipole^65–67^. To further demonstrate that the broad reflection dip is closely related to the excitation of a magnetic dipolar mode, we have also calculated the radiated power from electric dipolar moment *I*_***p***_, magnetic dipolar moment *I*_***m***_, electric quadrupole dipolar moment *I*_***EQ***_, and magnetic quadrupole dipolar moment *I*_***MQ***_, by using the equations of conventional multipole expansion^68^. As clearly seen in Fig. 4 that, at the broad reflection dip the radiated power *I*_***m***_ is far larger than the radiated power *I*_***MQ***_, and is also several times of *I*_***p***_ and *I*_***EQ***_. This proves that indeed the magnetic dipole contribution is the dominant one.

**Figure 5:**
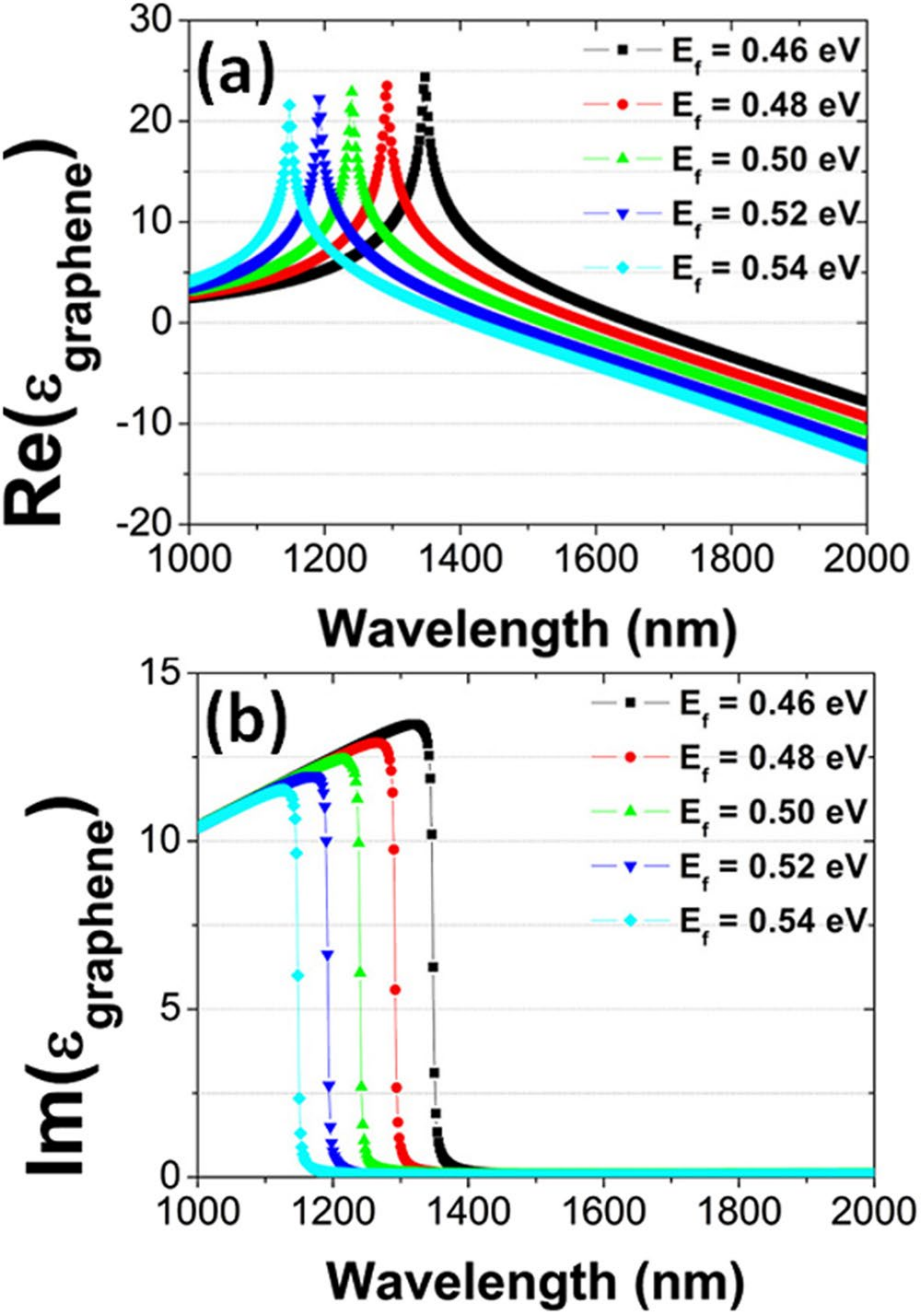
The real (**a**) and imaginary (**b**) parts of the effective permittivity *ε*_*g*_ of graphene as a function of wavelength, with Fermi energy *E*_*f*_ varied from 0.46 to 0.54 eV in steps of 0.2 eV. The other parameters are kept fixed.

When a bias voltage is applied to graphene, Fermi energy *E*_*f*_ will be changed, and thus the effective permittivity *ε*_*g*_ of graphene is electrically tunable. Figure 5 presents the dependence of the real and imaginary parts of *ε*_*g*_ on *E*_*f*_. For each value of *E*_*f*_, there is a sharp peak of the real part of *ε*_*g*_ in Fig. 5(a), which is related to the narrow interband transition in the monolayer graphene. Correspondingly, the real imaginary part of *ε*_*g*_ exhibits a deep drop for the wavelength to be increased, as clearly seen in Fig. 5(b). Such spectral properties can be easily shifted from 1348 to 1148 nm, when *E*_*f*_ is varied from 0.46 to 0.54 eV.

**Figure 6:**
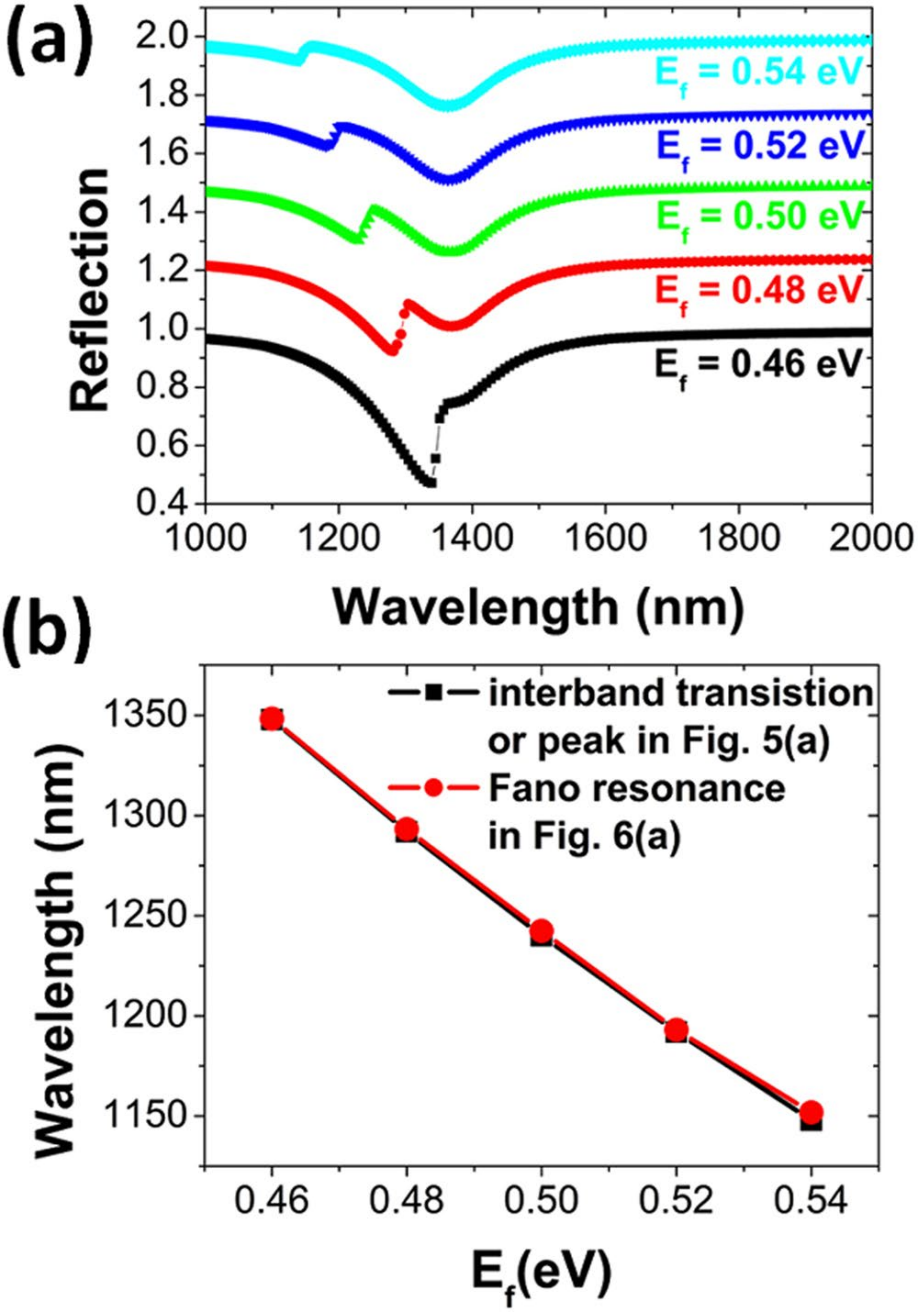
(**a**) Normal-incidence reflection spectra of metamaterials schematically shown in Fig. 1, with Fermi energy *E*_*f*_ varied from 0.46 to 0.54 eV in steps of 0.2 eV. The other parameters are kept fixed. For clarity, individual spectra are vertically offset by 0.25 from one another, respectively. (**b**) The positions of Fano resonance and interband transistion as a function of *E*_*f*_.

The narrow interband transition in the monolayer graphene, manifesting itself as a sharp peak of the real part of *ε*_*g*_ in Fig. 5(a), is strongly dependent of Fermi energy *E*_*f*_. Therefore, the Fano resonance, arising from the coupling between the narrow interband transition and the broad magnetic dipole in metamaterials, can be electrically tuned by applying a bias voltage to graphene to change *E*_*f*_. To demonstrate this, we have calculated a series of reflection spectra of metamaterials for different *E*_*f*_. As clearly seen in Fig. 6(a), the position of Fano resonance will red-shift and its asymmetric line shape will become more pronounced, when *E*_*f*_ is continuously decreased to shift the narrow interband transition to be much closer to the broad magnetic dipole. In Fig. 6(b), we have also compared the positions of the interband transition and Fano resonance. Obviously, they are completely overlapped in positions, further confirming that the Fano resonance is indeed the result of the coupling between the interband transition and the magnetic dipole.

**Figure 7:**
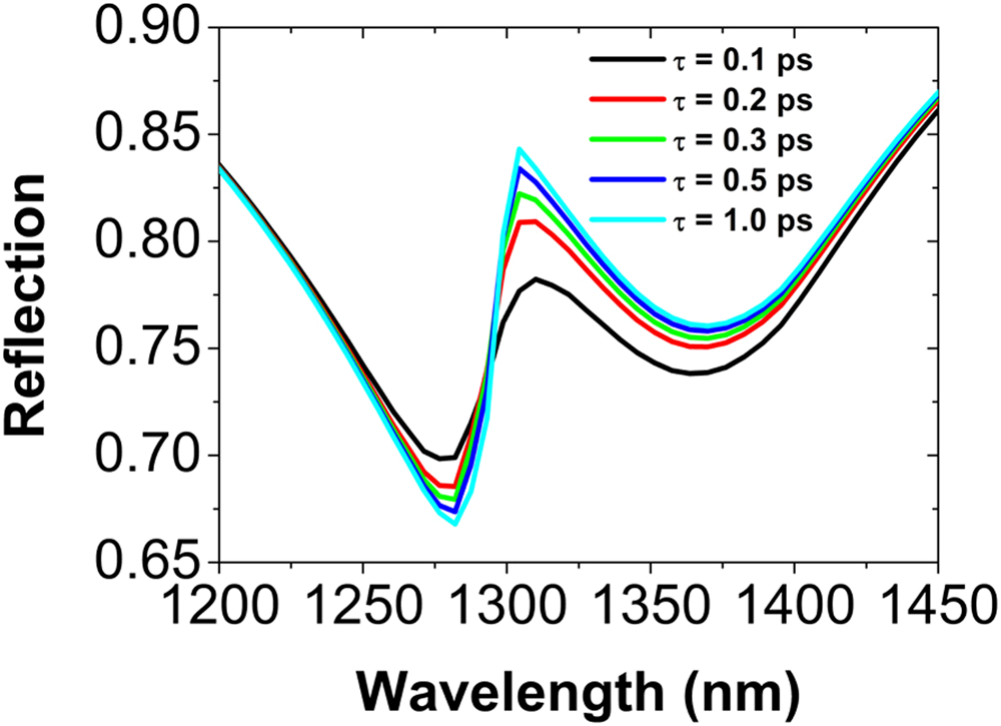
Normal-incidence reflection spectra of metamaterials schematically shown in Fig. 1, with the relaxation time of electron-phonon *τ* = 0.1, 0.2, 0.3, 0.5, and 1.0 ps. The other parameters are kept fixed.

We have also studied the effect of the relaxation time of electron-phonon *τ* on the Fano resonance. In Fig. 7 we present a series of reflection spectra of metamaterials for different *τ*. When *τ* is increased from 0.1 to 1.0 ps, the Fano resonance will become more sharp and pronounced. The underlined physics is that, with increasing *τ*, the imaginary part of the effective permittivity *ε*_*g*_ of graphene is decreased (increased), and thus the light absorption in graphene becomes weak (strong) at the peak (dip) of the Fano resonance.

Finally, we would like to do some discussions on the above numerical results. Naturally occurring materials usually have the saturation of the magnetic response beyond the THz regime. In recent years, exploring artificial metamaterials with various subwavelength building blocks to achieve a strong magnetic response has being drawing a lot of interest. The sharp Fano resonance associated with a magnetic field enhancement may find potential applications in magnetic nonlinearity and magnetic sensing. In a recent work, higher than 60% modulation in the transmission coefficient is reported at near-IR frequencies, by electronically tunable Fano resonance in a hybrid graphene/dielectric metasurface^60^. In our work, the wavelength of the Fano resonance can be conveniently shifted by varying the Fermi energy *E*_*f*_. The strength of the Fano resonance in the reflection spectra can also be tuned by changing the relaxation time of electron-phonon *τ* in graphene. As pointed out in ref.^60^, such a Fano resonance in the near-IR frequency range would have promising applications, such as biosensing and amplitude modulator.

In conclusion, we have numerically investigated the interesting phenomenon of Fano resonance in metamaterials, consisting of a metal nanodisk array on graphene, a dielectric spacer, and a metal substrate. The near-field plasmon hybridization between individual metal nanodisks and the metal substrate results into the excitation of a broad magnetic dipole. There exists a narrow interband transition dependent of Fermi energy *E*_*f*_, which manifests itself as a sharp peak of the real part (or an abrupt change of the imaginary part) of the effective permittivity *ε*_*g*_ of graphene. The strong coupling between the narrow interband transition and the broad magnetic dipole leads to the appearance of Fano resonance. The Fano resonance can be electrically tuned by applying a bias voltage to graphene to vary *E*_*f*_. The position of Fano resonance will be shifted obviously and its asymmetric line shape will become more pronounced, when *E*_*f*_ is varied for the narrow interband transition to progressively approach the broad magnetic dipole.

## Method

### Simulation

In this work, the reflection and absorption spectra, and the distributions of electromagnetic fields are calculated by “EastFDTD, version 5.0”^61^. In our numerical calculations, two perfectly matching layers (PML) in the *z*-axis direction are applied to eliminate the boundary scattering, and periodic boundary conditions are used for the *x* (*y*)-axis directions. For the graphene monolayer with a thickness of 0.35 nm, the minimum mesh size is set to be 0.05 nm. In the other calculated region, a refined homogeneous mesh size of *Δs* = 2 nm and a time step of *Δt* = *Δs*/2*c* (*c* is light speed in vacuum) are set manually to ensure numerical convergence. To obtain the reflection and absorption spectra, a Gauss pulse with a center wavelength of 1400 nm is utilized as a light source. The distributions of electromagnetic fields at a plane can be recorded conveniently by using this software package.
